# Alternative Splicing Regulation in Metabolic Disorders

**DOI:** 10.1111/obr.13950

**Published:** 2025-05-27

**Authors:** Dorota Kaminska

**Affiliations:** ^1^ Department of Medicine, Division of Cardiology, David Geffen School of Medicine University of California, Los Angeles Los Angeles California USA

**Keywords:** alternative splicing, dyslipidemias, metabolic dysfunction‐associated Steatotic liver, obesity, type 2 diabetes, weight loss

## Abstract

Alternative splicing (AS) is a fundamental mechanism for enhancing transcriptome diversity and regulating gene expression, crucial for various cellular processes and the development of complex traits. This review examines the role of AS in metabolic disorders, including obesity, weight loss, dyslipidemias, and metabolic syndrome. We explore the molecular mechanisms underlying AS regulation, focusing on the interplay between *cis*‐acting elements and *trans*‐acting factors, and the influence of RNA‐binding proteins (RBPs). Advances in high‐throughput sequencing and bioinformatics have unveiled the extensive landscape of AS events across different tissues and conditions, highlighting the importance of tissue‐specific splicing in metabolic regulation. We discuss the impact of genetic variants on AS, with a particular emphasis on splicing quantitative trait loci (sQTLs) and their association with cardiometabolic traits. The review also covers the regulation of spliceosome components by phosphorylation, the role of m6A modification in AS, and the interaction between transcription and splicing. Additionally, we address the clinical relevance of AS, illustrating how splicing misregulation contributes to metabolic diseases and the potential for therapeutic interventions targeting splicing mechanisms. This comprehensive overview underscores the significance of AS in metabolic health and disease, advocating for further research to harness its therapeutic potential.

## 1 Introduction

Alternative splicing (AS) was discovered in 1977 by Richard J. Roberts and Phillip A. Sharp, who identified it as a new mRNA synthesis mechanism in adenovirus 2 [1, 2]. Their discovery of “split genes” earned them the Nobel Prize in Physiology or Medicine in 1993. AS of the immunoglobulin gene in mammals was first observed in 1980 [3, 4]. Subsequent research has shown that this process is widespread among multicellular eukaryotes, including humans, with recent estimates indicating that up to 95% of human multi‐exon genes undergo AS [5, 6].

While genetic polymorphisms (SNPs) and gene expression have traditionally received more attention, AS gained prominence following the unveiling of the human genome map in 2001, which revealed a significantly lower number of protein‐encoding genes than previously thought. With only around 20,000–25,000 protein‐encoding genes [7]—far fewer than the previously estimated 120,000 genes [8]—but an estimated one million proteins in humans [9], AS has been recognized as a crucial mechanism for generating proteomic and regulatory diversity in higher eukaryotes.

**FIGURE 1:**
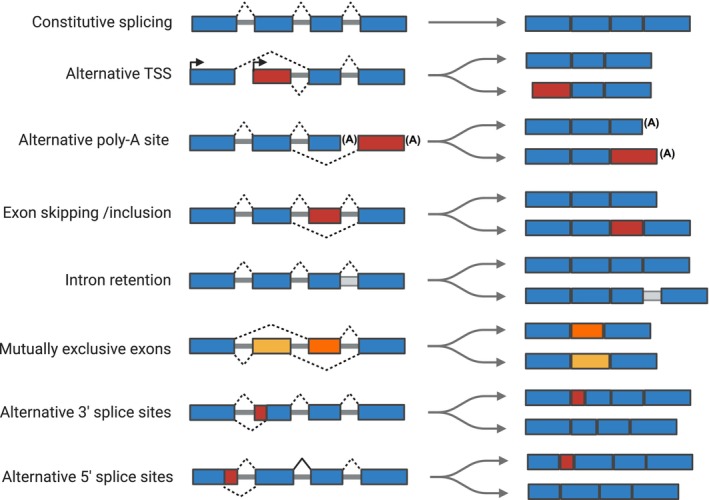
Different types of alternative splicing. In the diagram, exons are depicted as boxes while introns are illustrated with solid lines, and dashed lines represent potential splicing alternatives. Constitutive exons are colored blue, alternative exons are red, and mutually exclusive exons are shaded yellow and orange. Transcription start sites are marked with arrows, and polyadenylation sites are denoted by the symbol (A). Created in BioRender. Kaminska, D. (2025) (https://BioRender.com/d10k390).

**FIGURE 2:**
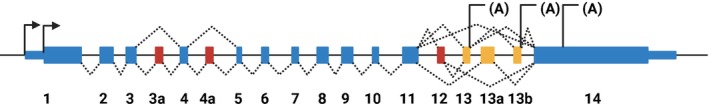
Alternative splicing of *TCF7L2* gene. Constitutive exons are colored blue, alternative exons are red, and mutually exclusive exons are shaded yellow and orange. Transcription start sites are marked with arrows, and polyadenylation sites are denoted by the symbol (A). Created in BioRender. Kaminska, D. (2025) (https://BioRender.com/v73i842).

AS of pre‐mRNA serves as a common regulatory mechanism that modulates both gene transcripts and cellular protein content. There are seven types of AS events, such as variations in transcription start sites (TSS), exon utilization, poly‐A site selection, exon skipping, intron retention, mutually exclusive exon usage, and splice site selection at the 3′ and 5′ ends (Figure 1). These splicing events can occur across different exons within a gene and may vary between tissue types. For instance, the *TCF7L2* gene demonstrates various functional splicing variations (Figure 2), including alternative TSS, exon 3a and 4a skipping [10], and mutually exclusive splicing of exons 13, 13a, and 13b, which are tissue‐specific. Exon 13 is expressed in adipose tissue, exon 13a in the liver, and exon 13b in the pancreas [10, 11, 12, 13]. The inclusion of alternative 3′ end exons 12, 13, 13a, and 13b leads to alternative poly‐A site utilization [14].

Regulation of transcript abundance primarily occurs at the transcriptional level while AS dictates the composition of transcripts and their encoded proteins. This regulation influences various aspects such as protein localization, activity levels, interactions between proteins, binding properties, oligomerization tendencies, and posttranslational modifications [15]. On average, each gene produces approximately 10 alternatively spliced variants [16].

## 2 Spliceosome Assembly

The catalysis of splicing within the nucleus is facilitated by a dynamic ribonucleoprotein complex known as the spliceosome. The spliceosome comprises five small uridine‐rich ribonucleoproteins (snRNPs: U1, U2, U4, U5, and U6) and over 170 distinct proteins [17]. Specific sequence elements found in each exon are recognized by spliceosomal components: the 5′ splice site (5′SS), the 3′ splice site (3′SS), and the branch site (BS). The BS is typically located 18–40 nucleotides upstream from the 3′SS and is followed by a polypyrimidine tract (PPT).

The canonical mechanism of splicing is well understood. The process begins with the U1 snRNA binding to the 5′SS, followed by the subsequent recruitment of the U2 auxiliary factor (U2AF) to the PPT and 3′SS, binding of U2 snRNP to the BS, recruitment of the U4–U5–U6 complex leading to replacement of U1 by U6. Conformational changes result in dissociation of the U1 and U4 snRNPs while associating U2 and U6 form a lariat intron eventually removed. The U5 snRNP aids in merging the two exons (reviewed in [18]).

**FIGURE 3:**
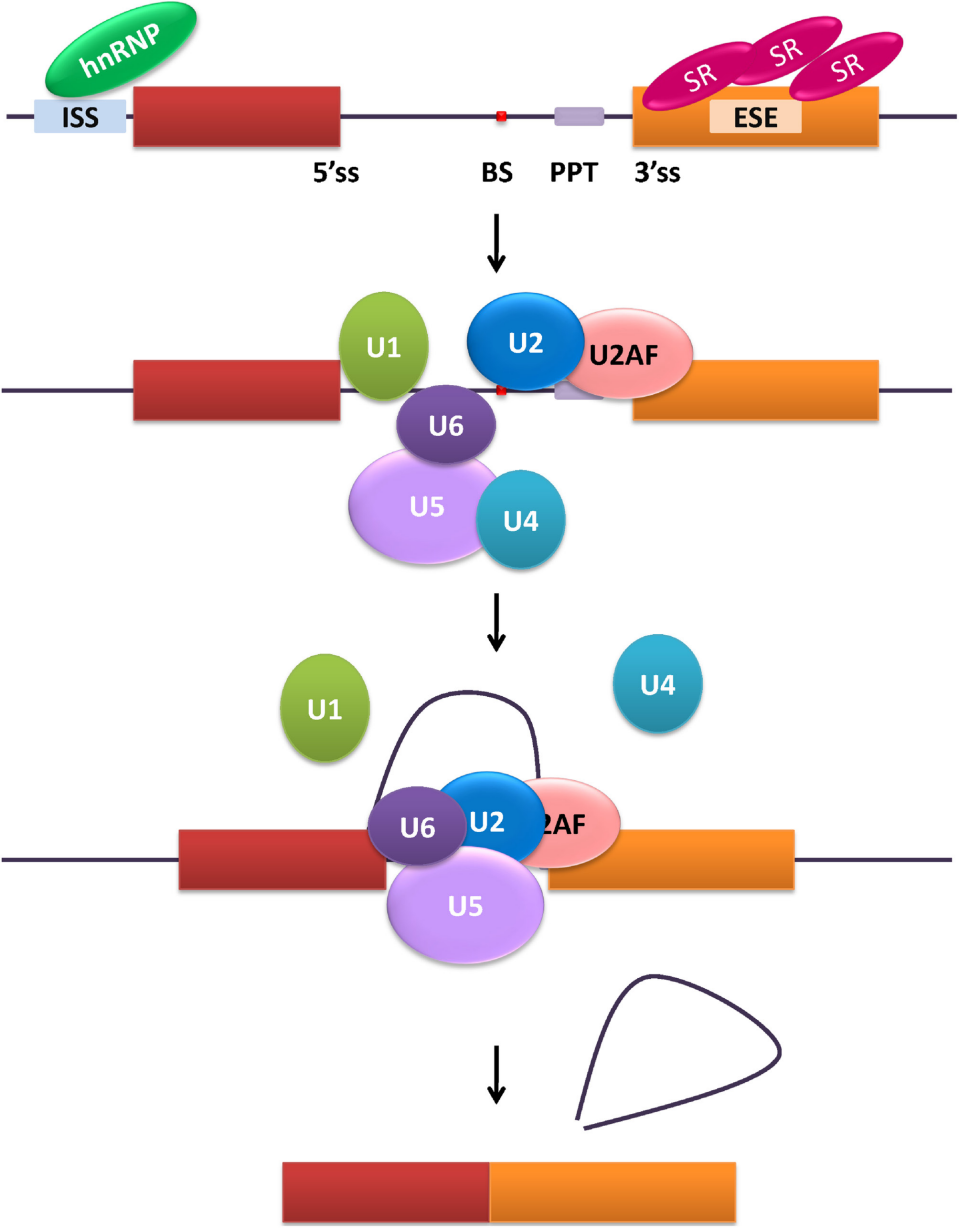
Splicing and spliceosome assembly. Pre‐mRNA splicing, carried out by the spliceosome, involves sequential binding and release of snRNPs and numerous protein regulators.

A brief overview of the splicing and spliceosome assembly process is illustrated in Figure 3.

## 3 Mechanisms of AS Regulation: *Cis*‐Acting Elements and *Trans*‐Acting Factors

AS is orchestrated by the interplay between *cis*‐acting elements within pre‐mRNA and *trans*‐acting RNA‐binding proteins. *Cis*‐acting elements, such as exonic and intronic splicing enhancers and silencers (ESE, ISE and ESS, and ISS, respectively), serve as binding sites for *trans*‐acting factors, influencing spliceosome assembly and exon recognition. These *trans*‐acting proteins, including SR proteins and heterogeneous nuclear ribonucleoproteins (hnRNPs), modulate exon inclusion or exclusion by interacting with *cis*‐acting elements and influencing splice site selection. SR proteins bind to splicing enhancers, promoting U2AF/U2 recruitment at the 3′SS and U1 snRNP at the 5′SS, while hnRNP proteins typically bind silencers to block snRNP or SR protein binding [18]. However, SR proteins at intronic sites can inhibit splice site recognition, whereas hnRNP A/B/C/F/H binding at introns can enhance splicing [19].

The regulation of splicing is highly complex and depends on the relative levels of both types of *trans*‐acting proteins, resulting in the selection of exons included in the final transcript through the combined action of antagonistic regulatory proteins. Interestingly, many of the *trans*‐acting factors are often expressed as alternatively spliced isoforms and participate in cross‐regulation and self‐regulation [20].

Tissue‐specific AS is believed to result from differences in the expression of *trans*‐acting factors. For instance, SRSF3 has been shown to be crucial for functional hepatocyte differentiation, hepatic lipogenesis, and cholesterol synthesis [21].

Recent advances in genomics have highlighted the significant role of splicing quantitative trait loci (sQTLs) in the regulation of AS. sQTLs are genetic variants that affect the splicing of pre‐mRNA, either by influencing the recognition of splice sites or by modulating the activity of splicing enhancers and silencers. These variants can be categorized into *cis*‐sQTLs and *trans*‐sQTLs. *Cis*‐sQTLs are located near the gene they affect and directly influence splicing by altering the binding of splicing factors or the splicing machinery. Mapping *cis*‐sQTLs is relatively straightforward due to their strong linkage with local genes. GTEx has successfully mapped *cis*‐sQTLs in metabolic tissues by leveraging RNA‐seq data from up to a few thousand individuals. Interestingly, the GTEx project showed that 23% of GWAS loci overlap with sQTLs and 43% with eQTLs, highlighting the role of splicing in gene regulation. Most sQTLs affected UTRs, influencing RNA stability and translation efficiency [22]. *Cis*‐sQTL were also successfully mapped in over 400 adipose tissue samples collected in the METSIM study [23], identifying 203 sQTLs in human subcutaneous adipose tissue. These sQTLs colocalized with 141 genome‐wide association studies (GWAS) signals for cardiometabolic traits. This included 25 signals for lipid traits, 24 signals for body mass index (BMI), and 12 signals for waist‐hip ratio adjusted for BMI [23].

*Cis*‐sQTLs are well‐documented in metabolic diseases, impacting key genes involved in glucose homeostasis [24, 25], lipid metabolism [23, 26], and obesity [23]. It has been reported that sQTLs in islet cells impact genes essential for islet function and diabetes management, with several sQTLs directly linked to T2D genetic variants. Notably, T2D has been associated with an sQTL that creates a nonsense isoform in *ERO1B*, a key regulator of ER stress and proinsulin biosynthesis [27].

In contrast, *trans*‐sQTLs remain significantly less explored, primarily due to the complexity of their regulatory mechanisms and the larger sample sizes required to detect them with confidence. Mapping *trans*‐sQTLs, presents a significant challenge due to their subtle effects, complex long‐range interactions, and potential confounding by population structure. Large sample sizes, often ranging from tens to hundreds of thousands of individuals, are necessary to achieve sufficient statistical power for accurate *trans*‐sQTL mapping. Large biobanks, such as the UK Biobank and FinnGen, combined with deep RNA sequencing data and sophisticated statistical modeling, offer promising resources for identifying trans‐sQTLs and unraveling their roles in complex traits and diseases. Furthermore, short sequencing reads can misalign due to sequence similarities, significantly affecting *trans*‐eQTL analyses. Over 75% of *trans*‐eQTLs detected by standard pipelines are likely false positives from misalignment, particularly among similar genes [28]. This concern is also expected to affect *trans*‐sQTL analyses. *Trans*‐sQTLs, act through splicing regulatory factors that are not located near the gene they influence. These can include splicing factors that are themselves regulated by genetic variants, affecting the splicing of multiple target genes across the genome. Analysis of immune cells from 105 healthy individuals identified i2‐rQTLs (integrated isoform ratio QTLs) that influence protein structure and downstream transcriptional effects. The study revealed that protein‐altering sQTLs can regulate gene networks via *trans*‐eQTL effects, potentially acting as causal mechanisms for GWAS loci linked to complex diseases [29].

The identification of sQTLs has provided insights into the genetic basis of splicing regulation and its impact on various diseases.

## 4 Regulation of Spliceosome Components and *Trans*‐Acting Factors by Phosphorylation

The activity of both spliceosome components and *trans*‐acting factors is modulated through reversible phosphorylation and dephosphorylation events. Numerous studies have highlighted the critical role of phosphorylation in activating spliceosomal components [30, 31, 32].

Regulative phosphorylation and dephosphorylation events apply also to SR proteins, causing a physiologically important change of splicing pattern. Phosphorylation enhances the SR protein's spliceosome recruitment, localization, and RNA‐binding affinity [33]. For example, insulin activated Akt phosphorylates SRSF5, this phosphorylation modulates splicing of *PKCβ* leading to the exon inclusion, production of *PKCβII* isoform and glucose uptake stimulation [34, 35], while TNFα induced dephosphorylation of SRSF5 leads to exon skipping and generation of *PKCβI* isoform, which does not facilitate glucose uptake [36]. Insulin also increases phosphorylation of SRSF1, SRSF2, and SRSF3 in hepatocytes, whereas arachidonic acid reduces phosphorylation of SRSF3 and SRSF5 by 50%–80% [37], potentially modulating metabolic responses.

## 5 The Role of m6A Modification in AS

m6A methylation, the most common posttranscriptional modification, has been shown to regulate AS via m6A‐binding proteins [38, 39]. m6A marks near splice sites recruit splicing factors, influencing whether exons are included or skipped. The role of m6A regulators in RNA metabolism and several examples of their functions in glycolipid metabolic disease has been detailed in a review [40].

Polymorphisms in m6A regulators impact splicing and contribute to disease susceptibility. The *FTO* gene, strongly linked to obesity in humans, regulates splicing via its m6A demethylation function [41, 42, 43]. *FTO* demethylates, exon 6 of *Runx1t1*, promoting its skipping and resulting in the production of the shorter, adipogenesis‐promoting isoform, *Runxt1*‐*S* [44]. The profile and level of *FTO* expression vary depending on which variant of the single nucleotide polymorphism (SNP) is present (reviewed in [45]). Understanding m6A‐splicing interactions may reveal new therapeutic targets for metabolic diseases.

## 6 RNA Polymerase II in AS Regulation

AS and transcription, are tightly coupled, with splicing machinery recruited to pre‐mRNA before RNA polymerase II (RNA pol II) completes transcription. Promoter choice and RNA pol II elongation rates influence exon inclusion, potentially driving cell‐specific metabolic regulation [46].

Two distinct models have been proposed to elucidate RNA pol II's involvement in AS: [1] the recruitment model, where binding of SR proteins to the carboxyl terminal domain of RNA pol II modulate exon inclusion/skipping [47] and [2] the kinetic model where faster RNA pol II elongation favors exon skipping and constitutive exon inclusion while slower elongation promotes alternative exon inclusion [48].AS.

## 7 Histone Modifications and Chromatin Structure in AS Regulation

Posttranscriptional histone modifications, such as methylation, acetylation, and phosphorylation, in regulating AS. Epigenetic modifications and noncoding RNAs regulate AS, influencing metabolic gene expression. Histone methylation slows RNA pol II, promoting exon inclusion, while acetylation enhances RNA pol II processivity, leading to exon skipping (reviewed in [49]). These mechanisms are relevant to metabolic regulation, as seen in T2D‐associated CpG‐SNPs affecting *SLC30A8*, *WFS1*, *CDKAL1*, and *TCF7L2* splicing in pancreatic islets ASAS [50].

Additionally, noncoding RNAs such as microRNAs, siRNAs, and *MALAT*‐*1* have emerged as regulators of AS patterns [48]. MicroRNAs can regulate the expression of splicing factors such as splicing repressor PTB [51]. siRNAs have been suggested to modulate chromatin structure near alternatively spliced gene regions [52], and the long noncoding RNA MALAT‐1 sequesters inactive SR proteins within nuclear splicing speckles. Diminishing MALAT‐1 levels leads to heightened activity of SR proteins, ultimately promoting the inclusion of specific exons in genes [53]. These findings highlight epigenetic and RNA‐based regulation as key players in metabolic gene expression.

## 8 AS Conservation Across Species

Although tissue‐specific gene expression patterns are generally conserved across mammals, AS is only partly conserved. Gene expression profiles across species tend to cluster by tissue type, indicating that a given tissue's transcriptome (e.g., liver) remains largely consistent across species. However, AS profiles cluster more by species than tissue, suggesting that AS changes evolve more rapidly and play a significant role in species differentiation [54]. While some highly tissue‐specific AS events are preserved across mammals, especially in brain, heart, and muscle, often linked to essential functions like phosphorylation or protein–protein interactions, other tissues like liver, lung, kidney, and spleen show species‐dominated AS patterns [54]. Furthermore, rhesus‐specific AS events were shown to be twice as likely to be shared with humans than rodent‐specific events. This suggests that while the core splicing machinery is preserved, the specific splicing events can diverge during evolution due to lineage‐specific functional requirements.

Despite differences in species‐specific AS profiles, the core splicing machinery and regulatory networks remain conserved, making animal studies instrumental in uncovering sQTLs relevant to human diseases. Animal models provide a valuable system for identifying functionally relevant splicing variants that may also be conserved in humans. A recent study of bone mineral density (BMD) in diversity outbred (DO) mice identified 732 genes with significant sQTLs with significant sQTLs in mesenchymal cells. Notably, sQTLs in *Fgfrl1* and *Tpx2*, genes regulating bone formation, colocalized with human BMD‐associated loci, demonstrating the cross‐species relevance of sQTLs in complex diseases [29]. Interestingly, the extent to which sQTLs contribute to complex traits and disease heritability appears to vary across species. While in humans, both eQTLs and sQTLs explain only a small fraction of trait and disease heritability [55], in cattle, they jointly account for the majority of trait heritability [56]. This vast difference is likely driven by the lower genetic diversity resulting from highly controlled selective breeding in livestock, longer linkage disequilibrium blocks that facilitate variant detection, and differences in environmental contributions, with livestock experiencing more controlled conditions that reduce environmental noise in heritability estimates. These findings highlight the evolving role of sQTLs in complex traits and diseases across species. While their impact varies, further research comparing human and animal models will be essential to better understand their contribution to disease heritability.

## 9 Measuring AS

Conventional methods for exploring AS involve techniques such as Northern blot and reverse transcription polymerase chain reaction (RT‐PCR) from a complementary DNA (cDNA) library, followed by electrophoresis. The RT‐PCR approach allows for precise detection of individual exons. Although effective for single‐gene analysis, these methods are inadequate for high‐throughput assessments, posing a notable constraint.

Advancements in high‐throughput transcriptome evaluation technologies over the past decade have facilitated comprehensive exploration of AS patterns on a genome‐wide scale. Three primary sources of transcriptome data have been leveraged for investigating splicing patterns: expressed sequence tags (ESTs), high‐density exon‐junction microarrays, and RNA sequencing (RNA‐seq).

ESTs are derived from sequencing short cDNA fragments. Aligning EST contigs with genome sequences provides insights into various transcript variants and polymorphisms. Nonetheless, ESTs offer only fragmented transcript details and may propose hypothetical transcripts not expressed in vivo [57].

High‐density exon‐junction microarrays utilize oligonucleotide probes to target known exons and exon‐junctions. However, this method lacks information about sequence and gene structure and necessitates prior knowledge of existing splice variants [58].

RNA‐seq has emerged as a potent approach for studying AS by generating millions of short sequence reads from one or both ends of sequenced fragments, producing extensive, highly sensitive transcript data [59]. Unlike microarrays, RNA‐seq does not mandate prior knowledge of transcript variants or genome sequences, facilitating the detection of novel splice junctions and exonic sequences. Nevertheless, deciphering RNA‐seq data, especially for genes with numerous alternative transcripts, present challenges due to the complexity of information.

The emergence of long‐read sequencing, capable of generating reads of 10,000 bases or more, has revolutionized genomic studies. This technology has enhanced read mapping accuracy, enabled de novo genome assembly, and improved the detection of structural variations, including repetitive elements [60, 61, 62]. A recent study utilizing long‐read RNA sequencing on 29 immune cell subsets uncovered a vast complexity of splicing events, significantly influenced by the insertion of transposable elements (TEs) into the human genome. This study also highlighted cell type‐specific AS in immune cell subsets, underscoring the need for single‐cell splicing analyses over bulk analyses. Additionally, the study identified 84 novel genes with isoform fractions significantly altered between individuals with systemic lupus erythematosus (SLE) and healthy individuals and revealed that sQTLs are enriched in loci associated with complex diseases, including immune‐mediated diseases and neurological disorders [63]. These findings underscore the potential of long‐read sequencing to uncover novel splicing events that could serve as therapeutic targets or biomarkers for various diseases and indicate that we are only beginning to understand the full extent of splicing complexity.

Computational advancements allow quantification of AS events from RNA‐seq data using four main approaches: (1) splice event‐based, (2) known isoform‐based, (3) exon‐based, and (4) junction‐based. Splice event‐based methods (e.g., rMATS [64], MAJIQ [65], and SUPPA [66]) quantify specific splicing events using percentage spliced in (PSI) that analyze the specific events as shown in Figure 1. Isoform‐based methods (e.g., Cuffdiff2 [67] and SUPPA [66]) estimate differential transcript usage but require accurate isoform annotations. Exon‐based methods (e.g., DEXSeq [68]) quantify differential exon usage but require predefined exon annotation. Junction‐based methods (e.g., LeafCutter [69]) analyze exon–exon connectivity and are ideal for novel junction detection, too. Integrating multiple approaches enhances splicing analysis accuracy and biological relevance. Tools like LeafCutter [69] and sQTLseekeR [70], THISTLE [55], or GLiMMPS [71] were designed to identify sQTLs by correlating splice junction usage with genotype data.

## 10 Functions of AS

The fundamental role of AS is to enrich transcriptome diversity and expand the genome's coding capacity. Despite its importance, the functions of many alternatively spliced exons remain unknown. Although most AS events occur in coding regions [72], variations in UTRs also affect translation efficiency, as seen in insulin mRNA, where a 5′UTR variant alters insulin synthesis and secretion, leading to hyperinsulinemia [73].

Furthermore, AS can shed light on the roles of certain synonymous SNPs. An A‐to‐G substitution in the *ACADM* gene, which disrupts a binding site for hnRNPA1 and creates one for SRSF1 without altering the amino acid sequence, modifies fatty acid oxidation [74]. Splicing can also modify metabolic enzyme function of products from different genes. For example, *FADS1* splice variants that encode proteins with medium reading frames enhance FADS2 desaturation activity, increasing eicosanoid precursor production [75]. It is noteworthy that up to one‐third of alternative transcripts undergo nonsense‐mediated decay to prevent their translation [76].

AS plays a crucial role in facilitating rapid metabolic adaptation to changing environments. Nutrients and hormones regulate SR proteins' activity in hepatocytes, which modulate the splicing of the glucose 6‐phosphate dehydrogenase (*G6PD*) gene [37]. Under conditions like starvation and high‐fat diets, impaired intron removal from the *G6PD* transcript reduces lipogenesis [77].

## 11 AS in Regulation of Glucose and Lipid Metabolism

Numerous genes related to glucose and lipid metabolism are regulated by AS. Table 1 contains examples of genes with known isoform function.

**TABLE 1:** Functional consequences of alternative splicing.

Gene	Splicing event	Function	Reference
*INSR*	Alternatively spliced exon 11	INSR‐A—cell growth; INSR‐B—glucose homeostasis	Belfiore et al. [78]
*LPIN1*	Alternatively spliced exon 6	LPINα—proliferation; LPIN1β—lipogenesis	Peterfy et al. [79]
*ACBP*	Several splice variants	ACBP‐1C—lipogenesis	Ludewig et al. [80]
*TCF7L2*	Several splice variants	TCF7L2 isoforms exhibit different ability to activate Wnt signaling in adipose tissue and distinct effect on pancreatic beta cell turnover.	Weise et al. [81], Le Bacquer et al. [82]
*PPARγ*		PPARγ2—higher ability to induce adipogenesis than PPARγ1	Mueller et al. [83]
*Pref*‐*1*	4 different splice variants	Pref‐1A and Pref1‐B inhibit adipogenesis whereas Pref‐1C and Pref‐1D do not affect adipogenesis	Mei et al. [84]
*HMGCR*	Alternatively spliced exon 13	HMGCR13(−)—reduces statin sensitivity	Yu et al. [85]
*LDLR*	Several splice variants	Attenuated LDLR activity	Kulseth et al. [86]
*GPD1*	Splice variant consisting of 3 exons and retained intron	Increased secretion of triglycerides	Basel‐Vanagaite et al. [87]
*GIPR*	Splice variant retaining intron 8	Dominant negative effect against wild‐type GIPR	Harada et al [88]
*FADS1*	Several splice variants resulting in proteins with short, medium and long reading frames	FADS1AT1—enhanced FADS2 activity	Park et al. [89].
*FADS2*	Alternatively spliced exons 1, 2, 3, 4	FADS2AT1—changed fatty acids profile	Reardon et al. [90]
*FADS3*	Several alternatively spliced isoforms	Unknown function	Park et al. [91]
*ACOX1*	Mutually exclusive exon 3a or 3b	ACOX1a—lower activity toward palmitoyl‐CoA than ACOX1b	Vluggens et al. [92]
*CETP*	Alternatively spliced exon 9	CETPΔ9—inhibited secretion of full‐length CETP	Quinet et al. [93]
*mTOR*	Truncated variant terminated within intron 5	mTORi5—inhibited adipogenesis	Huot et al. [94]
*Pax4*	Alternatively spliced exon 7	Pax4v impaired suppression of the insulin promoter	Lin et al. [95]
*Isl1*	Alternative 3′ splice site within exon 5	Isl1β—more potent stimulation of insulin promoter activity than Isl1α	Ando et al. [96]; Lin et al. [95]
*PKM*	Mutually exclusive exon 9 and 10	PKM1—facilitates energy production through glycolysis; PKM2—regulates aerobic glycolysis	Morita et al. [97]

## 12 Nuclear Receptor Splicing and Its Impact on Metabolic Diseases

Nuclear receptors (NRs) are ligand‐activated transcription factors that play a crucial role in regulating various metabolic processes and signaling pathways. Numerous splice variants have been described for these metabolic receptors, as reviewed in [98]. These isoforms contribute to the complexity of metabolic regulation and offer potential targets for therapeutic interventions in metabolic diseases such as diabetes, metabolic syndrome, and fatty liver disease, indicating the complex role of splicing regulation in metabolic health and disease.

## 13 AS in Diseases

Given the complexity and prevalence of AS, it is not surprising that its misregulation plays a significant role in various diseases. AS can be disrupted by point mutations (SNPs), which have been documented in various databases [99, 100, 101, 102]. It is estimated that at least 10% of SNPs are located within canonical splicing signals, and approximately 25% of missense mutations alter splicing. Overall, recent estimates suggest that around one‐third of all disease‐causing mutations affect mRNA splicing [19, 103, 104]. Moreover, these estimates do not account for SNPs located within intronic acting elements, which can also impact splicing. Aberrant splicing that leads to disease can result from point mutations within acting elements or from the misregulation of the splicing machinery due to environmental factors. A substantial body of literature reviews the roles of acting mutations in diseases, illustrating the critical impact of splicing misregulation on health (reviewed in [105, 106, 107, 108, 109, 110, 111, 112, 113]).

## 14 Role of Acting Mutations in Metabolic Dysregulation Disorders

Table 2 shows splicing mutations generating aberrantly spliced transcripts.

**TABLE 2:** Splicing mutations generating aberrantly spliced transcripts.

Phenotype	Gene	Mutation	Clinical manifestation	Molecular manifestation	Reference
Obesity	*PHF6*	A>G substitution in intron 2	Mild Börjeson–Forssman–Lehmann syndrome	Exon 3 skipping	Vallee et al. [114]
	*GNB3*	c.825C>T	Obesity	Truncated protein	Siffert et al. [115]
	*LEPR*	G>A in the splice donor site of exon 16	Early‐onset morbid obesity and pituitary dysfunction	Truncated protein lacking both the transmembrane and the intracellular domains	Clément et al. [116]
	*LRRFIP1*	rs11680012 (C)	Abdominal adiposity and inflammation	Potentially altered exonic splicing	Plourde et al. [117]
Obesity/hyperinsulinemia/hypertriglyceridemia	*ALMS1*	IVS18‐3T4G	Alström syndrome	Exon 19 skipping and truncated protein	Sanyoura et al. [118]
Dyslipidemia	*LDLR*	rs688 (T)	Familial hypercholesterolemia	Decreased exon 12 splicing efficiency due to mutation within ESE	Zhu et al. [119]
		G186G; R385R		Truncated exon 4; truncated exon 9	Defesche et al. [120]
		c.1187‐10 G>A		Premature termination codon, reduced LDLR activity	Holla et al. [121]
		c.2140+86 C>G		Retained insert from intron 14	Kulseth et al. [86]
		c.940_940+14del15		Deletion of 15 nucleotides resulting in impaired protein function	Maglio et al. [122]
		rs72658867 (A)	Lowers non‐HDL‐C	Retention of intron 14 resulting in truncated LDLR	Gretarsdottir et al. [123]
	*GPD1*	c.361−1G>C	Transient childhood hypertriglyceridemia and fatty liver followed by hepatic fibrosis	Truncated protein	Basel‐Vanagaite et al. [87]
	*APOB*	c.1471‐G>A; c.3697‐1G>C	Familial hypobetalipoproteinemia	Truncated proteins, not secreted to the plasma causing severe fatty liver	Di Leo et al. [124]
	*AGPAT2*	c.493‐1G>C	Congenital generalized lipodystrophy (extreme reduction of adipose tissue)	Mutation in splicing acceptor site, skipping of exon 4 essential for acyl‐CoA transferase activity	Cortés et al. [125]
	*MTTP*	c.619G>T; c.1237‐28A>G	Abetalipoproteinemia (low lipid levels and absence of apoB‐containing lipoproteins)	Truncated protein, incapable of binding protein disulfide isomerase	Pons et al. [126]
		c.1236+2T>G		Skipping of exon 9, nonfunctional truncated protein	Magnolo et al. [127]
		c.2342+1G>A		Partial retention of intron 16, nonfunctional truncated protein	
	*ABCA1*	c.1195‐G>A; c.4465‐4A>G	Tangier disease and familial HDL deficiency	Intron retention, truncated protein	Fasano et al. [128]
		c.2961‐2A>C		Truncated protein	Bocchi et al. [129]
		c.1510‐1G>A		Activation of a cryptic splice site in exon 13, premature stop codon	Maranghi et al. [130]
	*HMGCR*	rs3846662 (A)	Associated with LDL‐cholesterol levels and myocardial infarction	Exon 13 skipping, altered enzymatic activity	Burkhardt et al. [131]; Hiura et al. [132]
	*APOB*	c.3843‐2 A>G	Familial hypobetalipoproteinemia	Exon 25 skipping, premature termination codon	Di Leo et al. [133]
	*CETP*	c.544C>T; c.802C>T	Primary hyperalphalipoproteinemia	Nonfunctional truncated protein	Cefalu et al. [134]
		rs5883 (T); rs9930761(C)	Increased HDL cholesterol levels in males	Exon 9 skipping	Talmud et al. [135], Papp et al. [136]
	*DHCR7*	IVS5+3A>T	Smith–Lemli–Optiz syndrome	Insufficient DHCR7 activity during embryogenesis	Koo et al. [137]
	*HNRNP1*	rs1920045 (T)	Attenuated response to statins	Exon 8 inclusion	Yu et al. [85]
Energy production	*ACADM*	IVS3+5G>T	The fatty acid oxidation disorder	Intron retention causing a frameshift	Waddell et al. [138]
		c.1161A>G	Affects fatty acid oxidation	Synonymous mutation, leads to change in preferential binding of hnRNPA1 and SRSF1	Bruun et al. [74]
	*ABCD1*	c.1081+5G>C	X‐linked adrenoleukodystrophy	Exon 2 to 5 skipping	Amorosi et al. [139]
	*ACADVL*	c.879‐8T>A	Very long chain acyl‐CoA dehydrogenase deficiency	Intron retention	Shchelochkov et al. [140]
	*ACOX1*	c.636+471G>T	Defective peroxisomal fatty acids beta‐oxidation	Exon 4 skipping	Rosewich et al. [141]
Accumulation of triacylglycerol, droplets in most tissues	*ABHD5*	c.47+1G>A	Chanarin–Dorfman syndrome	Dramatically truncated protein leading to almost complete absence of protein associated with severe steatohepatitis	Redaelli et al. [142]
		c.960+5G>A		Truncated protein	
Hyperinsulinemia	*ABCC8*	c.1333‐1013A>G	Hyperinsulinemic‐hypoglycemia	Inclusion of an out‐of‐frame pseudoexon, premature stop codon	Flanagan et al. [143]
	*HADH*	c.636+471G>T	Hyperinsulinemic‐hypoglycemia	Inclusion of an out‐of‐frame pseudoexon, premature stop codon	Flanagan et al. [143]
	*INSR*	c.1610+1G>A	Type A insulin resistance syndrome	Loss of function	Jin et al. [144]
Hyperglycemia	*INSR*	A>G substitution in intron 4	Rabson–Mendenhall syndrome	Exon 5 skipping	Kadowaki et al. [145]
	*GCK*	IVS5‐1G>A	MODY, associated with a gestational diabetes	In frame insertion of 27 bp	Toaima et al. [146]
	*PAX4*	IVS7‐1G>A	MODY type 9	Activation of cryptic splice site in exon 8, deletion of glutamine at position 250 (Q250del)	Sujjitjoonet al. [147]
	*HNF*‐*1α*	IVS8nt+1G>A; IVS4nt‐2A>G; IVS7nt‐6G>A	MODY	Exon 8 skipping; exon 5 skipping; exon 7 skipping; truncated proteins	Bulman et al. [148]
	*G6PC2*	rs560887 (G); rs2232321 (G)	Elevated fasting plasma glucose	Exon 4 inclusion	Baerenwald et al. [149]
	*GIPR*	rs10423928 (A)	Decreased insulin secretion, increased risk of type 2 diabetes	Reduced expression of variant coding for fully active receptor	Ahlqvist et al. [150]
Metabolic Dysfunction‐Associated Steatotic Liver (MASLD)	*HSD17B13*	rs72613567 (A)	Higher liver steatosis, lower fibrosis and inflammation	Splice donor variant, truncated protein	Abul‐Husn et al. [151]
Dunnigan‐type familial partial lipodystrophy type 2	*LAMN*	c.1488+5G>C mutation at the intron 8	Diabetes with insulin resistance, hypertension and hypertriglyceridemia, partial lipodystrophy	Retention of intron 8, truncated protein	Morel et al. [152]

## 15 *Trans*‐Acting Factors Implicated in Diseases

While the list of *trans*‐acting splicing mutations leading to diseases is extensive and continuously expanding, the role of *trans*‐acting factors in disease pathogenesis is equally critical. Mutations and changes in the expression levels of *trans*‐acting splicing factors can impact a wide array of genes. Mutations within *trans*‐acting factors are often lethal due to their broad regulatory roles [106].

For example, mutations in splicing factors such as PRPF3, PRPF8, PRPF31, and RP9—components of the U4‐U5‐U6 snRNP complex‐are associated with autosomal dominant retinitis pigmentosa [153]. Similarly, mutations in several splicing regulatory proteins, including SF3B1, SRSF2, U2AF35, ZRSR2, SF3A1, PRP40B, SF1, and U2AF65, have been linked to myelodysplastic syndromes [154].

The regulation of AS is heavily influenced by the relative abundance of *trans*‐acting splicing regulators. Thus, it is not surprising that alterations in the expression of these factors are implicated in various diseases. For instance, the expression levels of certain *trans*‐acting splicing factors have been linked to obesity, such as SFRS10 [155] or NOVA [156]. Several splicing factors have been implicated in white, beige, and brown adipose tissue adipogenesis (reviewed in [157, 158]). Additionally, the acting regulator hnRNP I is known to be regulated by both glucose and sterol levels, demonstrating the complex interplay between metabolic signals and splicing regulation [159]. In normal‐glucose obesity, there is an upregulation of genes coding for components of the major spliceosome and various splicing factors in subcutaneous (SC) preadipocytes when compared to lean individuals. Conversely, the development of insulin resistance/type 2 diabetes (IR/T2D), whether in simple or morbid obesity, is associated with a general downregulation of these splicing genes in SC preadipocytes [160]. RBFOX1 was shown to influence glucose‐stimulated insulin secretion in β‐cells via splicing of Gelsolin, a calcium‐activated actin‐binding protein that enhances insulin secretion [161]. And RBM4 was reported to promote insulin gene expression by altering the isoform balance of the transcription factors Isl1 and Pax4 via AS control [95].

Recently, Arif et al. demonstrated that a deficiency of SRSF1, a canonical splicing factor, results in metabolic dysfunction‐associated steatohepatitis (MASH)‐like liver pathology and leads to global defects in the transcriptome [162]. The SRSF3 protein has been shown to degrade during lipid overload or MASLD in both human and mouse samples [163]. Correspondingly, RNA splicing in the early stages of MASLD is significantly enriched for targets of the splicing factor SRSF3 [164]. It has been shown that livers of patients with obesity with steatosis exhibit significant alterations in spliceosome components and splicing factors. Alterations in the expression of core spliceosome components (RNU2, RNU6, SF3B1, RNU6ATAC, and RNU4ATAC) and specific splicing factors (PTBP1, SRRM1, SND1, and SRSF2) have been linked to the development of hepatic steatosis in patients with obesity. Notably, the splicing factor PTBP1 (HNRNPI) is increased in the livers of these patients and plays a crucial role in regulating the splicing of genes essential for fatty acid and cholesterol metabolism, such as *FADS2*, *FADS3*, *LDLR*, *MVK*, *HMGCS1*, and *PSCK9* [165]. These examples underscore the significant impact that *trans*‐acting splicing factors can have on gene expression and disease, highlighting the importance of further research into their roles and regulatory mechanisms.

## 16 Regulation of AS by Body Weight and Diet

AS has been linked to obesity and weight loss in several studies. Reduced expression of various *trans*‐acting factors in the liver and skeletal muscle of humans with obesity suggests a common dysregulation of splicing in obesity. This pattern has also been observed in mouse models of diet‐induced obesity, where downregulation of the *trans*‐acting factor TRA2B led to increased lipogenesis through the altered splicing and expression of the lipogenic β isoform of *LPIN1* [155].

AS of troponin T (*TNNT3*) further illustrates the relationship between body weight and splicing. In rat skeletal muscle, the splicing pattern of *Tnnt3* is influenced by both natural and experimental variations in body weight. Weight gain results in reduced inclusion of exon 4 in *Tnnt3*, suggesting increased calcium sensitivity and force output in weight‐bearing muscles like the gastrocnemius. Obese rats showed a progressively impaired ability to regulate skeletal muscle molecular composition homeostatically [166]. These results support the hypothesis that RNA splicing functions as a responsive mechanism to environmental changes such as increased body weight, rather than a primary driver of obesity.

Regulation of the mechanistic target of rapamycin (*mTOR*) splicing has also been linked to body weight. Sam68‐deficient mice, which exhibit a lean phenotype, produce a truncated variant of *mTORi5* due to altered splicing [94]. Additionally, neuronatin (*NNAT*) expression is regulated by nutrients and leptin in the hypothalamus, with *NNAT* existing in two isoforms, *NNAT‐α* and *NNAT‐β*. Reduction of *NNAT‐β* expression was observed after gastric bypass surgery in mice, but not after diet‐induced weight loss, suggesting that differential splicing of *NNAT* may contribute to weight loss mechanisms [167]. Studies have shown that splicing of *TCF7L2* in adipose tissue and liver is influenced by obesity surgery‐induced weight loss. Specifically, downregulation of the short mRNA variant, which lacks exons 12, 13, and 13a, was observed in response to weight loss in subcutaneous fat and liver. The short *TCF7L2* mRNA variant is associated with hyperglycemia and impaired insulin action in adipose tissue [13].

Furthermore, regulation of insulin receptor (*INSR*) splicing has been linked to body weight. The *INSR*‐*B* mRNA variant, important for glucose homeostasis, was upregulated in response to weight loss induced by both diet and gastric bypass surgery. *INSR*‐*B* levels were strongly negatively correlated with insulin levels [168]. This supports the notion that AS serves as an adaptive mechanism in metabolic regulation.

Dietary influences on AS have also been reported. For instance, a high‐fat diet induces the inclusion of exon 10 in *tau* mRNA in the brains of female mice [169]. Additionally, mice fed a cafeteria diet exhibited reduced expression and AS of the insulin‐degrading enzyme (*IDE*) in the liver [170]. These findings suggest that dietary composition influences RNA splicing, which in turn regulates metabolic pathways contributing to metabolic changes, but does not necessarily serve as a direct cause of metabolic disorders.

These findings collectively underscore the significant impact of body weight and dietary factors on the regulation of AS, highlighting the intricate relationship between metabolic states and gene expression. RNA splicing appears to function as a mediator in the relationship between diet and metabolic disorders, rather than being a primary causal driver. On the other hand, diet and weight loss were shown to influence AS events in genes within obesity GWAS loci, highlighting gene–environment interactions. Specifically, dietary intervention‐induced weight loss altered *MSH5* splicing, while *TRA2B* and *BAG6* exhibited BMI‐dependent splicing regulation, underscoring the interplay between metabolic status and genetic regulation of splicing [171]. Dietary composition itself can influence RNA splicing patterns, which in turn regulate metabolic pathways affecting disease progression. These effects are further modulated by gene–environment (G × E) interactions, where genetic predisposition may determine an individual's sensitivity to diet‐induced splicing alterations and subsequent metabolic outcomes.

## 17 Clinical Relevance of AS

The emerging role of AS in disease and its regulation by environmental stimuli have opened new avenues for diagnosis, prognosis, and drug development. AS influences gene expression, protein function, and metabolic pathways, making it a promising target for precision medicine approaches in metabolic disorders.

Polygenic risk scores (PRSs) quantify an individual's genetic predisposition to disease. PRSs bridge basic research and clinical applications by enabling risk stratification, improving diagnostic precision, and guiding personalized interventions, such as predicting treatment responses in cardiovascular and psychiatric diseases [172]. Furthermore, PRS interacts with environmental factors, as demonstrated in obesity research where diet and weight loss influence splicing regulation in obesity‐associated genes, highlighting the importance of gene–environment interactions in disease risk and management [173].

Current PRS models primarily rely on a sum of the trait‐associated alleles across many genetic loci, weighted by their effect sizes estimated from a GWAS but overlook the impact of sQTLs. Given the increasing evidence that sQTLs contribute to disease risk, incorporating sQTLs could significantly enhance their predictive power. This refined approach would enable earlier disease detection by identifying individuals at high risk even before the onset of clinical symptoms. Furthermore, it would enable more accurate risk stratification, allowing for personalized interventions and targeted therapies, ultimately advancing personalized medicine.

Aberrant splicing is implicated in the side effects of conventional drugs. For instance, a polymorphism within the *CYP2D6* gene (rs16947) alters exon 6 splicing, leading to reduced enzyme activity and affecting the metabolism of 25% of clinically used drugs [174]. This highlights the potential for developing drugs that specifically target RNA molecules or RNA‐binding proteins to correct splicing defects.

Several substances can modify AS, often acting indirectly through mechanisms such as histone acetylation or phosphorylation of regulatory factors (reviewed in [175]). Direct binding of drugs to pre‐mRNA can also alter splicing. For instance, pyrvinium pamoate promotes the inclusion of an alternative exon in the serotonin receptor 2C, which plays a role in appetite control [176]. Metformin, one of the most commonly prescribed antidiabetic drugs, has been reported to affect AS and correct several splicing defects associated with myotonic dystrophy type I, by modulating AMP‐activated protein kinase (AMPK) signaling, which interacts with splicing factors and RNA‐binding proteins [177]. Additionally, metformin significantly inhibits the proliferation and migration of oral squamous cell carcinoma cells [178] and suppresses hepatocellular carcinoma through the regulation of AS of the *LGR4* gene [179].

## 18 Therapeutic Approaches

Given the role of AS in obesity‐related metabolic disturbances, targeted modulation of AS presents a promising therapeutic strategy for obesity‐associated metabolic disturbances. The ability to manipulate specific splicing events offers a unique opportunity to address the underlying molecular mechanisms driving obesity and its related metabolic complications. Several approaches are being explored to modulate AS for therapeutic benefit, primarily focusing on targeting either *cis*‐regulatory elements within pre‐mRNA or *trans*‐acting splicing factors that influence splicing decisions.

Over the last 10 years (2013–2023), there have been remarkable achievements in advancing therapeutic antisense oligonucleotides (ASOs) into clinical use. These short single‐stranded synthetic nucleic acid sequences can bind to pre‐mRNA and directly affect transcription and splicing events. By sterically blocking or enhancing the binding of splicing factors, ASOs can alter the production of specific protein isoforms. ASOs have revolutionized the treatment landscape for a variety of genetic, neuromuscular, and metabolic disorders, offering targeted therapeutic interventions that were previously unattainable. Their ability to modulate splicing and degrade disease‐related mRNA transcripts has resulted in the approval of multiple ASO‐based drugs by both the United States Food and Drug Administration (US FDA) and the European Medicines Agency (EMA). These drugs can be broadly categorized into two mechanisms of action: splicing modulators, which alter pre‐mRNA splicing, and mRNA degraders, which lead to targeted transcript degradation via RNase H‐mediated cleavage. Among the splicing‐correcting ASOs, US FDA has approved mRNA eteplirsen, golodirsen, viltolarsen, and casimersen [180], which promote exon skipping in the DMD gene, enabling the production of a partially functional dystrophin protein in patients with Duchenne muscular dystrophy. Nusinersen, another FDA‐approved ASO, enhances exon 7 inclusion in SMN2 pre‐mRNA, restoring functional survival motor neuron (SMN) protein levels in patients with spinal muscular atrophy [181]. On the other hand, some ASOs function through mRNA degradation rather than splicing modulation [182, 183, 184]. Among these, mipomersen plays a unique role by targeting *ApoB*‐*100* mRNA for RNase H‐mediated degradation, ultimately reducing low‐density lipoprotein (LDL) cholesterol in patients with homozygous familial hypercholesterolemia. This underscores the diverse potential of ASOs in treating metabolic diseases beyond splicing‐related conditions [185]. EMA has also recognized the potential of ASO therapies, approving volanesorsen, an ASO that targets *APOC3* mRNA, leading to RNase H‐mediated degradation and significant triglyceride reduction. Volanesorsen is currently used to treat familial chylomicronemia syndrome, hypertriglyceridemia, and familial partial lipodystrophy, all conditions closely linked to obesity and metabolic syndrome [186]. These findings suggest that ASOs could play a broader role in metabolic disease management, potentially extending to MASLD, insulin resistance, and type 2 diabetes. Although promising, ASO therapies face challenges such as the need for intensive treatment protocols, frequent injections, and high costs.

Preclinical studies have shown promise for ASOs targeting obesity‐related genes. ASO‐induced skipping of *INSR* exon 11 in MIN6 cells demonstrated that the INSR‐B isoform is protective by enhancing beta cell survival through stronger activation of Ras‐MAPK–ERK and PI3K‐mTOR signaling pathways [187].

CRISPR/Cas9 (clustered regularly interspaced short palindromic repeats–CRISPR‐associated protein 9) technology has made significant strides toward clinical application. The US FDA recently approved the first therapy involving this genome editing technology [188]. CRISPR/Cas9 has emerged as a powerful tool for studying and regulating RNA splicing. By precisely targeting and editing specific genomic sequences, CRISPR/Cas9 can modulate pre‐mRNA splicing, leading to the inclusion or exclusion of particular exons. This genome editing approach could potentially enable permanent repair of splicing defects. EDIT‐101 (NCT03872479) was the first human clinical trial aimed at removing the genomic sequence containing the CEP290 c.2991+1655A>G deep‐intronic variant and its associated pseudoexon to restore full‐length mRNA and protein. The results of this small phase 1–2 study indicated that this first‐of‐its‐kind experimental treatment for Leber Congenital Amaurosis was both safe and efficacious, supporting further research into in vivo CRISPR‐Cas9 gene editing to treat diseases resulting from splicing mutations [189].

Another strategy involves small molecule modulators, which can indirectly influence splicing by targeting splicing factors or associated signaling pathways. This approach offers advantages in delivery and bioavailability compared to ASOs. RNA interference therapies, such as siRNA and shRNA, represent a third avenue for modulating AS. These molecules can target specific splicing factors or pre‐mRNA transcripts, altering splicing patterns and potentially ameliorating obesity‐related metabolic disturbances, risdiplam is the most notable FDA‐approved example, providing a noninvasive oral alternative to ASO‐based treatments for SMA [190].

Despite the therapeutic potential of AS modulation, several challenges remain. Ensuring the specificity of these interventions is crucial to minimize off‐target effects. Efficient delivery of ASOs and other splicing modulators to target tissues also presents a significant hurdle. Furthermore, the long‐term safety and efficacy of AS‐targeted therapies require thorough evaluation. Nevertheless, ongoing research and technological advancements continue to refine these approaches, paving the way for novel therapies that address the complex molecular underpinnings of obesity and its associated metabolic disturbances. These advancements hold the potential to revolutionize obesity treatment by targeting the root causes of the disease.

## 19 Future Research Directions

We have only begun to uncover the complexity of AS in metabolic regulation. Most published studies on AS rely on standard short‐read RNA sequencing, with a sequencing depth of 30 to 60 million reads per sample. Future research should incorporate long‐read sequencing and/or significantly higher sequencing depths, estimated at 100 to 200 million reads per sample, to enhance the detection of novel isoforms. The rapid development of long‐read sequencing technologies now enables full‐length transcript identification, significantly improving the detection of functionally relevant isoforms. Integrating long‐read sequencing with ribosome profiling will clarify which alternatively spliced isoforms are actively translated, providing deeper insights into their functional relevance in metabolism [191].

Increasing read length and expanding sample sizes will also enhance the ability to study *trans*‐sQTLs, enabling the identification of distant genetic variants that influence splicing regulation across different genes and pathways. This will be crucial for dissecting the complex gene–environment interactions that shape metabolic phenotypes. Investigating diet‐induced AS changes and linking these to sQTLs will further refine our understanding of how gene–environment interactions contribute to metabolic disorders.

Although transcriptomic analyses have greatly advanced our understanding of how AS contributes to transcript diversity, they do not always accurately reflect the diversity of the proteome. Many alternatively spliced isoforms may serve as noncoding regulators of gene expression, vary in stability, or be inefficiently translated, underscoring the need for studies that focus on functional protein outcomes. Combining long‐read sequencing with ribosome profiling offers a promising approach to identifying actively translated isoforms, bridging the gap between splicing patterns and functional protein expression, ultimately guiding the development of more effective splicing‐based therapies. As research progresses, integrating multiomic approaches that combine genomic, transcriptomic, proteomic, and metabolic data will be essential to provide a deeper understanding of how AS influences metabolic pathways and establish a foundation for precision medicine strategies in managing metabolic diseases.

## 20 Conclusion

In conclusion, AS plays a crucial role in expanding the coding potential of the genome, thereby enhancing proteomic and regulatory complexity. Dysregulation of splicing is implicated in numerous diseases, highlighting the importance of understanding the underlying mechanisms. Advances in genomics have uncovered significant insights into the regulation of AS by genetic variants and environmental factors, paving the way for novel therapeutic strategies. The development of drugs targeting splicing mechanisms, including antisense oligonucleotides, holds promise for correcting splicing defects and treating various diseases.

Although we have made significant progress, we are only beginning to understand the full role of splicing in health and disease. With the advent of advanced technologies such as long‐read sequencing and single‐cell resolution analysis, we are likely to uncover even more about the intricacies of splicing. These technological advancements may enable the personalization of treatment approaches, offering more precise and effective therapies tailored to individual splicing profiles. Continued research into the roles and regulatory mechanisms of AS will be essential for furthering our understanding and improving disease management.

## Conflict of Interest

The author declares no conflicts of interest.
